# 

**Published:** 1918-02-16

**Authors:** 


Rates of Subscription: Twelve months, 6/6; Six months, 4/-; three months, 2/-, post free to any address in the United Kingdom. Abroad, Twelve months, 8/8; Six months, 5/-; Three months, 2/6, post free. **THE HOSPITAL "Index" to Volume LXII., April 1917 to September 1917, is now ready, price 2½d. per copy, post free.** Address The Manager, The Hospital, "The Hospital" Building, 28/29 Southampton Street, Strand, London, W.C. 2.

### DEATH OF A PROMISING PHYSICIAN.

The death, at the early age of thirty-seven years, of Dr. T. Arnold Johnston, removes from the medical life of Leicester a physician of ability and a student with many achievements. After gaining distinction at Belfast and Edinburgh Universities he filled several resident offices at the Royal Infirmary, Edinburgh, and became senior resident medical officer, and later assistant to the Professor of Clinical Medicine and Therapeutics. Subsequently he was appointed house physician to the Bradford Royal Infirmary, and later house physician at Leicester Royal Infirmary, and in succession hon. pathologist and consulting pathologist, and assistant physician there. His death is deeply regretted by a large circle of professional and other friends. A widow survives him.

### Gifts and Bequests.

Brigade-Surgeon James Davidson, of Aberdeen, has left £100 each to the Aberdeen Royal Infirmary and the Aberdeen Sick Children's Hospital, and £50 each to the Maternity Hospital, Aberdeen, and the Royal Medical Benevolent Fund.

Miss A. H. Cooke, of Muswell Hill, has left £200 to the Surgical Aid Society, £200 to the Bognor Convalescent Homes, and £100 to the London Temperance Hospital.

A number of Jewish charities benefit under the will of Mr. Simeon Lazarus, of the Stock Exchange, who also left £250 each to King Edward's Hospital Fund, the London, Guy's, University College, and Middlesex Hospitals, £200 to St. George's Hospital, £100 to the North London Hospital for Consumption, and the Ventnor Hospital for Consumption.

Mr. Louis Floersheim, of Cadogan Square, has left £2,000 to be distributed amongst hospitals and charitable institutions at the discretion of his executors.

Mr. Henry Shawe, of Wyke Cop Farm, Scarisbrick, Lancashire, divided the residue of his estate, amounting to about £5,000, between the Southport Infirmary and the "Comforts Fund" of the Ormskirk Military Hospital.

Mr. and Mrs. Alex. Cowpe, of Burnley, have given £1,500 to the Burnley Victoria Hospital for the endowment of three cots in the children's ward in memory of Lieutenant G. B. Cowpe, Cheshire Regiment, killed in action on July 31 last.

Mr. T. W. Bower, J.P., C.C., of Oxcroft, has given £500 to the Chesterfield Hospital, in memory of Second-Lieutenant Thomas G. Bower, Household Battalion.

Mrs. Agnes Johnson, of Stoneygate, who died on November 3 last, left £250 to the Maternity Hospital, Leicester; £100 to the Leicester Infirmary; £100 to the Faire Hospital, Leicester; £100 to the Leicester Children's Hospital; and £50 to the Leicester District Nursing Association.

### Passing Events and Topics.

### Farm Colony for Hull Consumptives.

Through the generosity of Sir James Reckitt, Bart., who has purchased a large residence and twenty-nine acres of land, to be let at a nominal rent to the Hull After-Care Committee, it has become possible to establish a farm colony for selected cases of tuberculosis at Walkington, near Beverley.

### Royal Army Medical College.

The Hygiene Department of the Royal Army Medical College has been removed from the University College, Gower Street, to Grosvenor Road, S.W., where all samples of foodstuffs, etc., submitted for analysis should be sent.

### Memorial to Mrs. Harley.

To perpetuate the memory of Mrs. Harley, who lived in Shropshire and had been prominently associated with the Woman's Movement in Shrewsbury for ten years, it has been decided to endow a Kathleen Harley Memorial ward in the Royal Salop Infirmary. The project has met with general approval, for, in the words of Queen Alexandra, "a more fitting tribute to the memory of this brave lady, who worked so unremittingly and unselfishly to alleviate the sufferings of the sick and wounded in this terrible war, and who made the great sacrifice while engaged upon her work of mercy, cannot be conceived."

### Manchester Police Gift of £10,000.

Lieut.-Col. C. J. Trimble, C.M.G., V.D., commanding the St. John Ambulance Brigade Hospital, Etaples, France, has been presented at Manchester with a cheque for £10,000, raised for the hospital through the efforts of the special and regular police forces of the city.

### Spotted Fever.

Eight cases of spotted fever have occurred at the Crystal Palace since December 31 last. All necessary steps are being taken.

The early publication by the house of Cassell is announced of new and enlarged editions of Treves and Keith's "Surgical Applied Anatomy" and Candy's "Manual of Physics." The former has been revised by Professor Keith, assisted by Dr. W. Colin Mackenzie, and the latter by Dr. Candy.

